# Epiviz File Server: Query, transform and interactively explore data from indexed genomic files

**DOI:** 10.1093/bioinformatics/btaa591

**Published:** 2020-07-03

**Authors:** Jayaram Kancherla, Yifan Yang, Hyeyun Chae, Hector Corrada Bravo

**Affiliations:** Center for Bioinformatics and Computational Biology; Institute for Advanced Computer Studies; Department of Computer Science, University of Maryland, College Park, MD 20742, USA; Department of Computer Science, University of Maryland, College Park, MD 20742, USA; Biology and Computer Science, Swarthmore College, Swarthmore, PA 19081, USA; Center for Bioinformatics and Computational Biology; Institute for Advanced Computer Studies; Department of Computer Science, University of Maryland, College Park, MD 20742, USA

## Abstract

**Motivation:**

Genomic data repositories like The Cancer Genome Atlas, Encyclopedia of DNA Elements, Bioconductor’s *AnnotationHub* and *ExperimentHub* etc., provide public access to large amounts of genomic data as flat files. Researchers often download a subset of data files from these repositories to perform exploratory data analysis. We developed Epiviz File Server, a Python library that implements an *in situ* data query system for local or remotely hosted indexed genomic files, not only for visualization but also data transformation. The File Server library decouples data retrieval and transformation from specific visualization and analysis tools and provides an abstract interface to define computations independent of the location, format or structure of the file. We demonstrate the File Server in two use cases: (i) integration with Galaxy workflows and (ii) using Epiviz to create a custom genome browser from the Epigenome Roadmap dataset.

**Availability and implementation:**

Epiviz File Server is open source and is available on GitHub at http://github.com/epiviz/epivizFileServer. The documentation for the File Server library is available at http://epivizfileserver.rtfd.io.

## 1 Introduction

Genomic data repositories like The Cancer Genome Atlas (The Cancer Genome Atlas Research Network *et al.*, 2013), Encyclopedia of DNA Elements (ENCODE; Davis *et al.*, 2018), Bioconductor’s (Huber *et al.*, 2015) AnnotationHub (Morgan *et al.*, 2019a) and ExperimentHub  (Morgan, 2019b) etc., provide public access to large amounts of genomic data as flat files. Researchers often download a subset of data files from these repositories to perform their exploratory data analysis. Increasing data size requires longer time to download, pre-process and load files into a database to run queries efficiently. This time-consuming process is an impediment to interactive analysis of genomic data.

Interactive visualization of data can be a powerful tool to enable exploratory analysis. As users get familiar with the data and gain insights, it would be even more efficient to interactively hypothesize, validate, visualize and compute the intermediate results of the analysis. Currently available interactive visualization tools for genomic data, namely genome browsers, fall into two broad categories. One that uses a database management system to load genomic data from files into tables, create indices or partitions for faster query of data by genomic intervals. The other category of genome browsers query data directly from indexed genomic file formats like BigBed, BigWig (Kent *et al.*, 2010) or Tabix (Li, 2011). However these tools are limited only to exploration of data from files.

NoDB is a new database design philosophy to make database systems more accessible and reduce the data-to-query time (Alagiannis *et al.*, 2012) by directly querying and transforming the raw data files instead of loading data from files into different storage, e.g. database tables. Based on these concepts, we developed Epiviz File Server (EFS), a Python library that implements an *in situ* data query and transformation system for local or remotely hosted indexed genomic files.

### 1.1 Contributions

Our design of the EFS library was based on the following goals:

1. Efficiently parse minimal necessary bytes from an indexed genomic file to query data for a specific genomic region.
2. Define transformations and summarizations directly over files and lazily compute these at query time.
3. Scale operations to concurrently process multiple file query and transformation requests.
4. Implement cache over files for faster access and improve repeat query performance
5. Provide REST API (Representational state transfer Application Programming Interface) for developers and bioinformaticians to build interactive visualization and exploratory tools over genomic data stored in flat files.
6. Integration with existing bioinformatic tools and software to interactively visualize and explore genomic data directly from files.

EFS decouples data retrieval and transformation from specific visualization and analysis tools and provides an abstract interface to define computations independent of the location, format or structure of the file. Our major contribution on this research project was to efficiently and intuitively define transformations and summarizations directly over files, without the hassle of downloading the files locally or pre-process for exploratory data analysis. Using the library, researchers and analysts can author shareable and reproducible data exploration workflows in an intuitive and programmatic way. EFS can query and explore data directly from local and publicly hosted indexed genomic files. If the files are hosted on a public server, the library requires the server hosting the data files to support HTTP range requests (https://tools.ietf.org/html/rfc7233), so that the parser can only request the minimum necessary byte ranges needed to process the query. The library supports various file formats - BigBed, BigWig and any tabular file that can be indexed using Tabix. Once these data files are described, users can define summarizations and transformations on these files using NumPy (or NumPy-like) functions (van der Walt *et al.*, 2011).

**Fig. 1.**
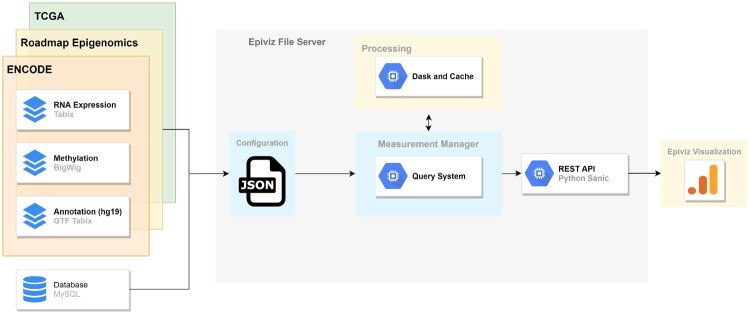
A high-level overview of the EFS Library. EFS library supports directly querying indexed genomic files. Data files are described in the measurements module and provides a programmatic interface to parse, query and define transformations over files using any NumPy(-like) function. Transformations are lazily computed at query time using Dask and the cache layer makes sure we only request for bytes not already accessed. Datasets and their transformations can be accessed using a REST API and allows developers to build interactive visualization and exploration tools

EFS uses Dask (Rocklin, 2015; https://dask.org) to scale and concurrently process multiple query and transformations over files. The cache implementation makes sure we only access bytes not already accessed and stored locally. Developers of bioinformatic tools and systems can use the library’s REST API to build interactive data visualization or exploratory tools over files. We demonstrate the integration in the use cases section of this paper by integration with Galaxy (Afgan *et al.*, 2018), a widely used open source bioinformatic Web platform for analysis of genomic data and with the Epiviz browser (Chelaru *et al.*, 2014), an interactive and integrative functional genome browser to visualize and explore these datasets. The browser supports various charts to explore genomic data, heatmap and scatter plots to visualize gene expression, block (linear and stacked) tracks for visualizing genomic regions of interest and line tracks (stacked, multi stacked) for visualizing signal (ChIP-seq, methylation etc.) data. Hovering over a region in one visualization highlights this region in other tracks providing instant visual feedback to the user. These visualizations are developed using standards-based Web component framework, are highly customizable, reusable and can be integrated with most frameworks that support HTML (Kancherla *et al.*, 2018). Figure 1 describes a high-level overview of these components in the EFS library.

### 1.2 Related work

Several existing genome browsers and tools support visualization of genomic data from flat files. These include the UCSC Genome Browser (Kent *et al.* 2002), Dalliance (Down *et al.*, 2011), JBrowse (Buels *et al.*, 2016), Integrated Genome Browser (Freese *et al.*, 2016). However, these tools only visualize genomic data from files and do not perform transformations over data. Integrated Genome Viewer (Robinson *et al.*, 2011) allows users to combine tracks through its interface and perform Add, Subtract, Multiply or Divide operations but they are tightly coupled to the visualization interface and not provided as a general-purpose library. RawVis (Bikakis *et al.*, 2018) converts user interaction queries into data access queries and dynamically builds an VALINOR (Visual Analysis Index on Raw data) index over the data attributes used in a specific visualization thus tightly coupling visualization and data workflows and is not specifically designed for genomic data files. HiGlass (Kerpedjiev *et al.*, 2018) is a visual genomic data exploration tool that supports several genomic file formats without re-indexing data and using existing zoom levels. HiGlass supports client-based divide by operations but other transformations are currently not supported by the system. WebWorkers can be accessed through JavaScript to asynchronously run computationally intensive tasks while keeping the UI in the browser responsive. However, limitations of the WebWorker thread model, namely that data are not shared across workers, applying transformations in the browser over large data may not be efficient. Computing transformations on the server allows multiple clients to access these results without having to individually compute them locally. EFS decouples data retrieval from visualization workflows, providing an interface to define transformations and a REST API to access them. Table 1 provides an overview of these features across related tools.

**Table 1.** Comparison of features across *in situ* file querying and transformation tools

Features	IGV	RawVis	HiGlass	UCSC	EFS
**Transformation across files**	Supports add, subtract, multiply and divide by operations.	Limited—computes data aggregations at query time for each dimension in a visualization.	Supports client-side divide by operations.	Does not support transformations across files.	Provides an interface to apply transformations across files using NumPy-like definition.
**Modularity**	Visualization and data workflows are coupled together.	Converts user interaction queries into data queries hence tightly couples visualization with data workflows.	Separates the user interface and the server.	Visualization and data workflows are coupled together in the browser.	Decouples data from visualization workflows, allows tools developers to develop tools using the REST API.
**Native** **support** **genomic** f**ile** **formats** (BigWig, BigBed, Tabix etc.)	Yes	No	Yes	Yes	Yes
**REST API to query files**	No	No	HiGlass-server provides an API to get tileset information and query by tiles.	REST API supports querying files from a TrackHub.	REST API supports querying both files and the transformations.

## 2 Materials and methods

### 2.1 *Traditional* approach

A naive approach to implement a query system over genomic files would be to first import the files into a database management system like MySQL. Accessing data from a database provides various advantages—we can query the data quickly by indexing on intervals (genomic position), we can use a standard declarative query language like SQL, and are able to modify the table schema on-the-fly. As the size of the data increases, there is a significant cost to initialize, load and prepare the dataset for queries. Our experience importing a histone modification data file from a ChIP-seq experiment into a database significantly increased the data-to-query time i.e. time to import the file into a table, time to index the table and time to query the table for a particular genomic region. Even after this, the database table had to be custom configured to use advanced indexing schemes like partitions and index by partition to improve the query performance. This is not a feasible approach to individually tune and optimize query performance when dealing with large public repositories like the NIH roadmap epigenomics (Bernstein *et al.* 2010) or ENCODE that host datasets from hundreds of experiments.

Assuming we do have all the data files imported into a database system, performing transformations on data directly is limited to the functions implemented in the system. Also to perform complex summarization or transformations across genomic datasets, we would need to first perform interval overlap operations that are limited or non-existent in database systems. To work around these limitations, one would implement backend programing logic (middleware) to first query the database for multiple datasets, perform interval overlap operations to align the data by genomic location, summarize and then apply transformations.

### 2.2 EFS approach

In the *traditional* approach, we expect the data-to-query time to significantly increase as the size of the data increases. The NoDB philosophy is a way of querying the data files directly, *in situ*, hence removing the loading time. A simple approach would be to load the data file into memory every time we need to query for a particular region. This is not efficient for (i) repeated query processing since we would load the same file into memory on every request and (ii) memory usage especially when querying several large data files at the same time. In order to work around the issue of loading the entire file into memory, we need to create and store an index based on genomic positions, query this index and only access the minimum necessary bytes of the file to process the query.

The genomics community has long used specialized binary file formats like BigBed, BigWig or Tabix for quickly accessing particular regions of the genome. These file formats contain an index to quickly and efficiently access blocks of the file that contain the data, then only parse these blocks to process the query. These formats also support remote file access, allowing multiple parallel requests to process at the same time. Our NoDB approach uses these indexed genomic file formats as the base for *in situ* query processing. Some of these file formats also summarize the data at different zoom levels (base-pair resolution) which is extremely useful especially for interactive exploration and visualization. A comparison of features between our approach and a traditional database server are listed in Table 2.

**Table 2.** Comparison of features between a traditional database server and EFS

Feature	Traditional DB (MySQL)	EFS
Time to query	Time to query is longer since the data needs to be imported into the database. Often requires custom indexing to improve query time.	EFS performs *in situ* query operations over indexed file formats.
Cache	Provides cache support for repeated query processing.	Implements cache for repeated query processing.
SQL	Provides an easy to use query language to search tables.	Does not provide a query language (but provides REST API).
Schema	Schema can be changed on the fly.	Changes in schema result in regenerating the index file.
Interval Overlap Operations	Does not provide interval overlap operations to apply transformations across datasets.	EFS supports overlap operations to apply transformations across files.
Transformations	SQL supports basic mathematical operations (average, min, max etc.) and often needs middleware to support any other transformations over query results.	EFS supports any transformation over the query results.

In addition to querying data from files, EFS provides an interface to compute transformations over data directly from files and is independent of the visualization interface. The library decouples data query and analysis from visualization workflows. This way new transformations can be applied over files and can be queried dynamically.

### 2.3 EFS library design and implementation

In this section, we specify the design and implementation of the EFS NoDB approach to querying and operating over genomic files.

#### 2.3.1 Parsing genomic file formats

EFS supports indexed genomic file formats—BigWig, BigBed, bam (with bai), sam (with sai) or any tabular data file that can be indexed using Tabix. These files either have an index as part of the same file (BigBed, BigWig etc.) or create a separate index file (Tabix). The index (usually a BTree or an RTree) allows the library to quickly navigate (or seek) and only access the blocks of the file that contains the data for a genomic region. These formats also support remote file access, allowing multiple parallel requests to process at the same time. We implemented data retrieval on these file formats in the *parser* module of the library. The library supports both remote and local file access and random-access queries. Developers can extend the base classes in this module to add parsers to other bioinformatic file formats. Results from the files are converted into a Pandas *DataFrame* (McKinney, 2010) using *IntervalIndex* to index intervals. This allows us to apply transformations, summarizations and other operations across files that contain different intervals.

#### 2.3.2 Defining and managing files

When accessing files from genomic data repositories that contain hundreds of files from various experiments, it is inefficient to individually describe these files. The *measurements* module in the library manages files within the library and implements several import functions to batch load data files from local or public repositories. To access files available through Bioconductor’s *AnnotationHub* or *ExperimentHub*, we implemented methods to directly import these into the library. The UCSC Genome Browser provides Track hubs (Raney *et al.*, 2014), a useful tool for visualizing large number of genome-wide datasets. Track hubs are web-accessible directories that contain genomic data and can be viewed on the Genome Browser. Methods are available to load track hub

from epivizfileserver import MeasurementManager

# create measurements manager

mMgr=MeasurementManager()

# add genome

genome=mMgr.add_genome( “ hg19 ” )

# i m port measurements from AnnotationHub

roadmap=mMgr.import_ahub(os.getcwd() +

**Fig. 2.**
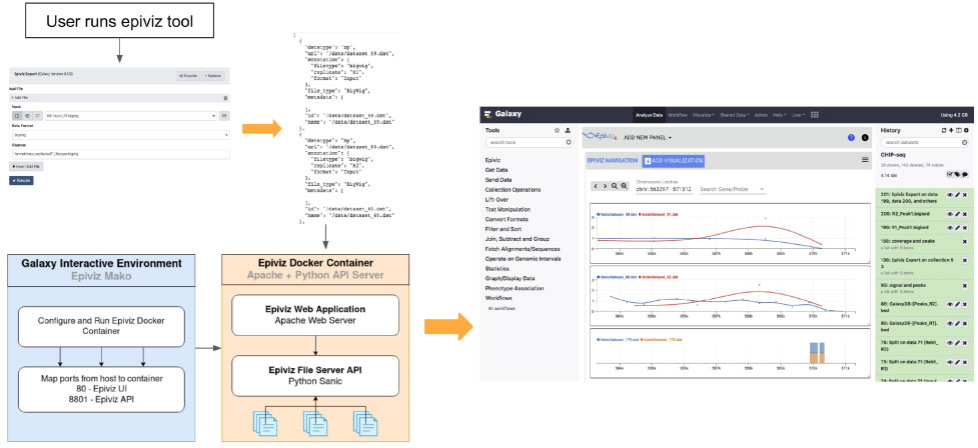
Overview of Epiviz integration with Galaxy. Users can include the Epiviz Galaxy Tool in a workflow to choose files and define annotations to generate an Epiviz configuration file. A Galaxy IE using the Epiviz configuration spins the Epiviz docker instance. Once the docker image loads, Galaxy embeds the user interface from the instance on its user interface as shown on the right

“ /roadmap.json ” )

repositories into the library. EFS library also supports a JSON-based configuration to define files. The configuration file defines a collection of files and for each file, describes the location of the file (public or full local path), its format, name, and annotations. An example configuration file is described in the use case section (Fig. 2). Regardless of how files imported, users can always define transformations over the files. The snippet below shows how one can import the files from *AnnotationHub*. One needs to create a measurements manager object which describes and manages various files available in the system. We also support a number of genomes (indexed using Tabix) for navigational queries and querying for annotations in a given genomic region.

#### 2.3.3 Transformations over files (computed measurements)

As previously stated, if an analyst needs to perform complex summarization or transformations across genomic datasets, they would need to first perform interval overlap operations that are limited or non-existent in database systems. To apply transformation across files that contain different intervals in a given genomic region, we should first find discrete intervals represented by the data, and then apply transformations over the overlapping regions. These interval operations and the transformation functions are limited in scope to the methods implemented in database management systems.

Existing genome browsers that support file-based visualization are only limited to exploration of data from files, i.e. query data from files and visualizing this data as tracks. As an illustrative example, if a researcher is exploring ChIP-seq data for a particular histone (*H3k24me3*) marker across different tissues, and would like to visualize the difference in binding across these tissues, the typical way is to use a computational environment or tools to read the files, align the data from these files to apply transformations, compute the difference and then store the dataset as a file or into a database. For the purpose of exploration, it would be very efficient to be able to define these transformations over files without having to pre-compute and interactively explore these transformations.

EFS allows defining transformations over files using methods available in NumPy or custom functions using a NumPy compatible definition (called *computed measurements* in the *measurements* module). These transformations are not pre-computed but lazily evaluated at query time. When querying these computed measurements for a particular genomic region, we first query the individual files for data, perform interval overlap operation to align the datasets and then apply the transformation. The snippet below describes how users can define computed measurements in the system. The first snippet shows how users can use an existing numpy function as a transformation; the other shows how to use a custom function that centers the signal around the mean as a transformation. The *computeFunc* parameter takes in the transformation you would like to apply to the files. The syntax is similar to the Pandas DataFrame *apply* function.

# filter for files from the measurements (brain samples)

brain=[“E071”, “E074”]

brain_files=out=[m for m in roadmap if m.id in [brain]]

# using Numpy, compute difference in binding

mMgr.add_computed_measurement(type=“computed”,

name=“Diff_Signal”, id=“Diff_Signal”, measurements=brain_files, computeFunc= numpy.diff, computeAxis = 1)

# custom transformation

def norm(col):

mean = numpy.mean(col)

return(col.apply(lambda x: x-mean))

mMgr.add_computed_measurement(type=“computed”,

name=“signal_norm”, id=“signal_norm”,

measurements=m, computeFunc=norm,

computeAxis = 0)

#### 2.3.4 Concurrency

When concurrent queries are made to the system, the *handler* module distributes these queries over available system resources. The *handler* module uses Dask, a lightweight distributed computing library for Python to scale and compute transformations. Dask manages, distributes and schedules tasks dynamically. The Dask scheduler is asynchronous and event driven, simultaneously responding to requests for computation from multiple clients. This provides flexibility to concurrently handle a variety of workloads from multiple users at the same time while also handling node failures and additions. The *handler* module is a wrapper around Dask to schedule queries to the system. The system automatically creates the handler when the application is run, but the user also has the flexibility to create a handler object and submit queries.

#### 2.3.5 Cache policy

To efficiently process queries, a traditional database server implements cache management and can provide scalability as the system demands. A file-based web server always has to access the files to query for a genomic region. This may not be efficient for repeated query processing for the same region. When querying BigWig and BigBed using the tools (*bigWigSummary* and *bigBedSummary*) from UCSC utilities (https://hgdownload.soe.ucsc.edu/admin/exe/) these tools implement a URL cache layer using the sparse file feature available in UNIX-based systems, which downloads and creates files locally as blocks of the files are accessed. One might quickly run out of space once you start caching hundreds of files from a repository locally.

EFS implements a simple cache management system to only store frequently accessed blocks as part of the file objects. The file objects always keep the header, zoom level and chromosome tree information as part of the file. This reduces the number of requests to the file (if available locally) or server hosting the file. As queries are processed, we store the binary blocks (or byte ranges) of the file that were recently and frequently accessed. The cache allows the library to quickly process repeated queries for a genomic region. As the number of queries and files increase, it also increases the cache size and impacts memory usage. The library implements task schedulers (through the *handler* module) that automatically serialize (pickle) file objects from memory to disk. This process efficiently manages memory and frees up system resources for other tasks. After storing a file object to disk, if a new request is made to query for a genomic region from the same file, we load (deserialize) the file object into memory and process the query. We should note that this simple cache expiration policy could be implemented by other tools to reduce space usage as well.

#### 2.3.6 Optimizations for visual analytics

EFS performs a number of optimizations mostly when querying for data from a file that contains zoom levels (BigWig and BigBed). This is extremely useful especially for interactive visualization and exploration. For example, visualizing signal data from a roadmap ChIP-Seq dataset for a 10 000 base-pair (bp) region, on a Webpage canvas of size 800 (width) by 400 (height) pixels. If the visualization library plots 1 bp per pixel, it would be very inefficient to try to render 10 000 data points in an 800-pixel width screen. In such scenarios, the library automatically chooses the appropriate zoom level to query for data, i.e. for the same example above, we choose the zoom level that has a reduction of around 12 bp per bin. If no zoom levels are available, the library also summarizes the data into smaller bins and computes a mean across each bin. This decreases the response to query time, improves rendering performance and is efficient for interactive visualization and data exploration for large genomic datasets. For example in the snippet below, the first query returns all intervals from the file whereas the second uses a zoom level (if available) and/or summarizes the data to 1000 bins.

# Query files

result err = await measurement.get_data(“chr11”, start = 10550488, end = 11554489)

result, err = await measurement.get_data(“chr11”, start = 10550488, end = 11554489, bins = 1000)

#### 2.3.7 REST API

All datasets loaded into the system can be easily queried using a REST API using the *server* module. The library uses Sanic (https://github.com/huge-success/sanic), an asynchronous library to make API queries to files and integrates well with the Dask system for handling Web requests. The REST API syntax is available in our documentation website.

## 3 Results

### 3.1 Use cases

#### 3.1.1 Integration with Galaxy

Galaxy (Afgan *et al.* 2018) is one of the widely used open source Web-based platforms for analysis of genomic data. Galaxy aims to make computational biology accessible to researchers with less programing experience. It has an easy to use user interface to create reproducible and shareable workflows and installs various bioinformatic tools without all the complexity. At its core, Galaxy is a file-based workflow platform, every step in the workflow creates a file(s) and these files are used as inputs in the next step. Our goal with integrating Epiviz with Galaxy is to create a single computational environment where users can analyze and explore datasets generated by Galaxy workflows. Galaxy provides Galaxy Interactive Environments (IEs), a framework to integrate external tools with Galaxy workflows and user interface.

To integrate Epiviz with Galaxy, we need to (i) Register and Run Epiviz as a tool with the Galaxy system, (ii) Define and access files generated at various steps in a Galaxy workflow and (iii) Query interactively visualize the files using Epiviz.

##### 3.1.1.1 Register and Run Epiviz with Galaxy

To integrate external tools, Galaxy provides a framework called Galaxy IEs (Grüning *et al.* 2017). The first step in IE is to create a docker container for Epiviz to manage its dependencies internally and also efficiently manage system resources. The docker container hosts both the EFS (the library to manage and query files) and the Epiviz user interface to interactively query and visualize datasets. IE requires a configuration file (mako) that spins up the docker instance on demand and run Epiviz inside Galaxy. The mako configuration file sets various parameters for the docker image and configures the ports to use from the image to serve the user interface. The library also needs access to the files generated during the Galaxy workflow. Instead of copying over files to the docker instance, we mount the data directory used by Galaxy to the docker image.

##### 3.1.1.2 Define files from Galaxy workflows

To define and access files to visualize using Epiviz, we created a Galaxy Tool (Blankenberg *et al.*, 2014) that the user integrates with their final step of the workflow. This tool allows the user to choose various files generated in their workflow, define annotations and file formats, and generates an Epiviz configuration file as shown in the top panel in Figure 2. EFS library running in the docker image from the previous step uses this configuration file to load various files into the instance.

##### 3.1.1.3 Visualize files in Galaxy workflow

After generating the configuration file in the previous step, users can visualize these datasets using Epiviz within the Galaxy interface. This displays the Epiviz application running inside the docker container on Galaxy and users can now visualize and interactively explore various files. The bottom panel in Figure 2 illustrates the process of integration and describes various steps in the process.

To demonstrate the integration, we use the Analysis of ChIP-seq data workflow from Galaxy (https://galaxyproject.org/tutorials/chip/). The workflow uses data where immunoprecipitation was performed with antibodies from Reb1.Reb1 recognizes a specific sequence (TTACCCG) and is involved in many aspects of transcriptional regulation by all three yeast RNA polymerases and promotes formation of nucleosome-free regions (Hartley and Madhani, 2009; Raisner *et al.*, 2005). After executing the entire workflow, users can now run the Epiviz tool to generate the configuration file on the coverage and peak calling files. The Galaxy screenshot in the right panel of Figure 2 shows the datasets visualized from the results of this workflow. The first track visualizes Replicate 1 and its Input signal, the second track visualizes Replicate 2 and its Input signal. After running the MACS2 peak calling tool on the dataset, the Galaxy workflow generates peak files and the last track visualizes these peak regions across both the replicates.

#### 3.1.2 Epigenome roadmap

The NIH Roadmap Epigenomics Mapping Consortium leverages next-generation sequencing technologies to map DNA methylation, histone modifications, chromatin accessibility and small RNA transcripts in tissues, selected to represent the normal counterparts of tissues and organ systems frequently involved in human disease. The data files from this consortium are available on the Roadmap Portal (http://www.roadmapepigenomics.org/).

**Fig. 3.**
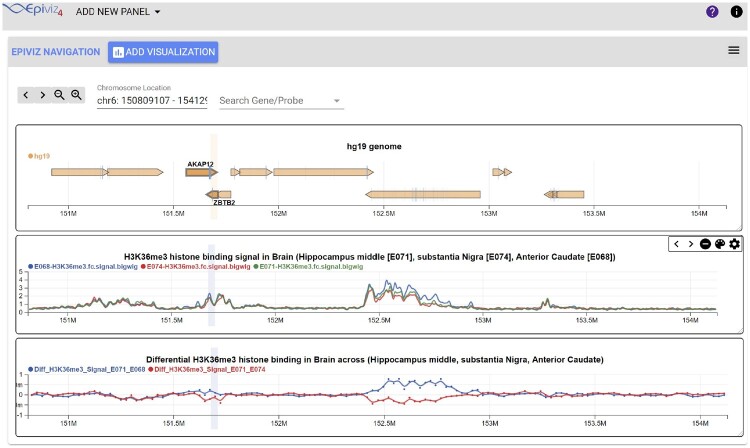
Interactive visualization of data from the NIH Roadmap Epigenomics project. This figure demonstrates the EFS library querying and computing transformations over data available from the NIH Roadmap Epigenomics project. We chose the ESR1 and its neighboring gene region for this example. (Top to bottom) The first track is a hg19 genome annotation track. The line track in the middle is visualizing the H3K36me3 binding signal from the ChIP-seq experiments across three different brain tissues. This track queries the data directly from the files. The last line track is a transformation over files to compute difference in histone binding across different tissues

For this use case, we use BioConductor’s *AnnotationHub* to query for data files that are part of the NIH Roadmap Epigenomics project. We downloaded the *AnnotationHub* SQLite database to extract information for all available resources in the hub. We then query this database for resources associated with the roadmap project. We get a total of 9932 data resources from *AnnotationHub*. These include DNA methylation signal, ChIP-seq fold change signal and *P*-values for various tissues and histone markers. To easily import these *AnnotationHub* records into the EFS, we added a helper function ‘*import_ahub*’. We filtered samples for the brain region and defined transformations over these files. We define difference in histone modification between different brain tissues for this dataset. We provide this file server instance as a public AMI (ID: ami-0de924a41fb56f52b) on Amazon Web Services (AWS) to explore the roadmap dataset and the transformations as shown in Figure 3. A short version of how this dataset is setup is illustrated in the code snippet below with a full example available in the documentation.

# create measurements

managermMgr=MeasurementManager()

# create a Dask handler

mHandler=create_fileHandler()

rfile=open(os.getcwd()+”/roadmap.pickle”, ”rb”)

roadmap=pickle.load(rfile)

# import measurements from AnnotationHub

roadmap=mMgr.import_ahub(roadmap, mHandler)

# filter for brain

datasetsbrain=[“E071”, “E074”]

froadmap=out=[m for m in roadmap

if m.id in [brain]]

# Apply transformations

mMgr.add_computed_measurement(“computed”,

”Diff_Signal”, ”Diff_Signal”, measurements=

froadmap, computeFunc=numpy.diff)

# Run the server with the measurements

app=setup_app(mMgr)

app.run(port = 8000)

### 3.2 Benchmarks

We performed several tests to evaluate (i) the performance with and without the cache implementation, and (ii) the overhead in lazily evaluating transformations. All tests were run on a standard Amazon AWS EC2 (t2.xlarge) instance with 4 vCPUs and 16 GB memory.

#### 3.2.1 Impact of cache on performance

To evaluate the impact of cache on the system, we randomly generated 20 different genomic range queries and repeatedly queried these against the Web server for 60 s. We use *wrk* (https://github.com/wg/wrk), a HTTP benchmarking tool capable of generating significant load to test the API. We run the tool on its default settings using 2 threads and 10 connections concurrently to send requests to the system.

We run the system on two different modes, one with the cache feature and the other without the cache. In the cache implementation, if the given genomic region already exists in the cache, it is fetched quickly and sent back to the user whereas in the non-cache setting, the library always parses the file to query data for the given genomic range. Since local file access is fast, our results are comparable between the cache and non-cache settings. Instead, we hosted the files on a S3 object store bucket at University of Maryland, adding network latency to the system. The results indicate the cache implementation significantly improves the performance of the system. Table 3 displays the results of these tests. To make sure other processes are not interfering in the benchmarking process, we disabled the serialization process for file objects discussed in Section 2.

**Table 3.** Impact of cache on processing requests

Implementation	Average Latency (in ms; ± SD)	Average Requests (per s; ± SD)
EFS—no Cache (remote file)	1152 (± 201.32)	8.2 (± 0.44)
EFS—cache (remote file)	68.41 (± 83.55)	179.4 (± 3.2)
EFS (local file)	36.05 (± 8.74)	284.1 (± 41.31)
PyBigWig (remote file)	121.864 (± 40.67)	—
PyBigWig (local file)	0.52 (± 0.28)	—

*Note*: This table displays the average latency and the requests processed per second measured when benchmarking the File Server API with and without a cache implementation. With cache, the library was able to process a significantly higher number of genomic range queries resulting in higher throughput and lower latency. The extra overhead in EFS is because of using intermediate data representations so that transformations can be performed across files and the use of JSON as a portable output format for multiple clients to query the system.

We also ran a similar experiment to compare the performance of EFS with existing bioinformatic tools. We use the PyBigWig python package (https://github.com/deeptools/pyBigWig), an extension to the C library, libBigWig that can read or parse local or remote BigWig and BigBed files. We run the same experiment as before, where we generate 20 random genomic ranges and execute these queries repeatedly for 60 s using the PyBigWig library. We read the same remotely hosted file and measure the average time per query and calculate SD. We notice that EFS performs significantly faster if the file is hosted remotely. Unsurprisingly direct access to the local file using PyBigWig is significantly faster compared with EFS. The extra overhead is EFS is due to the (i) use of an intermediate representation (Pandas *DataFrame*) so that transformations or summarizations can be performed across/within files and, (ii) using a portable data transfer representation of the results (JSON) so that multiple clients can query the system. PyBigWig only reports the intervals and the data in those intervals.

#### 3.2.2 Overhead in lazily computing transformations

EFS library lazily computes transformations or summarizations directly over files. We measure the overhead in computing transformations query time as opposed to pre-computing a transformation, storing and querying this file. For this test, we choose 20 different genomic datasets (bigwig files) from roadmap; we created an instance of the EFS that at query time computes a mean signal value with increasing number of files starting from 2 and up to 20 genomic datasets. In addition, we also pre-computed the mean signal using WiggleTools (Zerbino *et al.*, 2014) and store these files. This allows us to compare the overhead in on-the-fly computation versus the pre-compute.

**Fig. 4.**
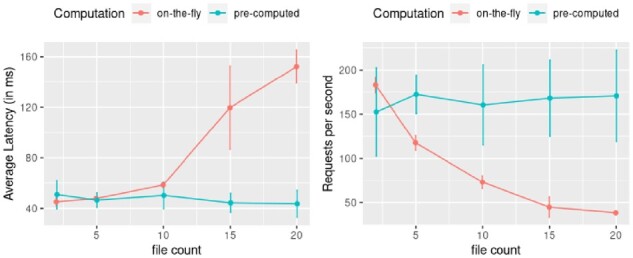
Impact of computing transformations at run time. We measure the overhead in computing transformations lazily (on-the-fly) versus querying a file that stores the pre-computed result. We measure the average latency and requests processed per second by the system across five different runs and the shows the mean and standard deviation for these metrics. The results indicate the latency of the system increases as we increase the number of files involved in the computation, hence lowering the number of requests processed per second

We run similar benchmarks as before using the *wrk* tool, where we randomly generate 5 different genomic range queries and query the system for 60 s (2 threads and 10 connections). We measure the average latency and requests processed per second, and calculate mean and SD of these metric across five different runs. Our results are shown in Figure 4. As expected, as the transformation involves more files, the latency of the system increases hence serving fewer requests per second compared with directly querying a pre-computed file. However, the system is still interactive with reasonable query response times.

### 3.3 Software availability

EFS is open source and is available on GitHub at https://github.com/epiviz/epivizFileServer. The documentation for the File Server library is available at http://epivizfileserver.rtfd.io. The package is published to PyPI and is available at https://pypi.org/project/epivizFileServer.

For integration with Galaxy, we have two different GitHub repositories, one for the Epiviz Tool (https://github.com/epiviz/epivizGalaxyTool) and the other for Epiviz IE (https://github.com/epiviz/epivizGalaxyIE). The repositories also contain instructions to setup the IEs in Galaxy.

For the use case describing the Roadmap dataset, the code is available in the GitHub repository inside the use cases folder. A tutorial of the library is also available at https://epiviz.github.io/post/2019-02-04-epiviz-fileserver/. We also provide an AWS image for this instance (AMI ID: ami-0de924a41fb56f52b).

A collection of snippets to quickly spin an instance of the File Server with publicly available data and interactively visualize using Epiviz browser is hosted on GitHub gists at https://gist.github.com/jkanche/1cd32ad8b2af9d59c834508ddc468359.

The code for benchmarks is also available in the same repository in the benchmarks folder.

The code is available under MIT License.

## 4 Discussion

EFS is a Python library to interactively query and transform data directly from indexed genomic files. The library implements several features provided by a traditional database system to query, transform, cache, scale and visualize the data from files. The library decouples data from analysis workflows and provides an abstract interface to define computations independent of the location, format or structure of the file. Because of this modularity in the implementation developers can use the library or the REST API to develop interactive genomic data visualization and exploration tools. This way new transformations can be applied over files and can be queried dynamically. With the integration of the Epiviz browser (but not limited to), EFS provides a quick way for researchers to perform exploratory visual data analysis directly over files.

The library currently works with indexed genomic file formats like BigBed, BigWig or Tabix. Most genomic data analysis workflows and pipelines generate BigWigs or BigBeds and most public repositories provide genomic data from experiments in these file formats. These formats support random access, concurrent and remote queries without the need to download or read the entire file into memory. The EFS library is an approach to implement a scalable data query and transformation system directly over these indexed files. EFS does not support offline conversion of files into the indexed format. If the files are available remotely, the server hosting the files must support HTTP range requests. This allows the library to only requests the minimum necessary bytes to perform the query. Most modern Web and FTP servers support range-requests.

Network latency is an important variable to consider when applying transformations over remote files. If the library is used to apply complex transformation over large number of remote files, we recommend running EFS with local access to files. This reduces both networks latency and speeds up query time. Our tests from the Benchmark section shows the performance of the File Server and the extra overhead in using the EFS library compared with existing bioinformatic tools like PyBigWig that can directly access local/remote BigWig/BigBed files. The extra overhead is due the use of an intermediate representation (Pandas *DataFrame*) to be able to apply transformations and a portable output format JSON to transfer data across the network for multiple clients. Even with the extra overhead, the system is highly performant and interactive. The PyBigWig library only returns the intervals and data from the file and to perform any analysis on top of these results would require extra processing of the data into a usable format.

The system works well for moderate sized repositories to apply complex transformations over files at query time. This depends on available system resources (processors, memory) for Dask to scale and the location of files (local or remote). Our tests on transformations show that latency of the system increases as we apply transformations over increasing number of files. The *parser* module spends the majority of time reading the index tree to find positions within the file that contain the data. For example applying a transformation over 20 files, the system has to individually parse the index of the 20 files to get the data and then apply transformations.

In addition to the usecases detailed above, Epiviz File Server is used to interactively query epigenetic data shared to the Neuroscience Multiomic Archive (NEMO, https://www.nemoanalytics.org/) and the gEAR portal (https://umgear.org/). These applications for visualization and analysis of multiomic data use Epiviz and EFS to visualize and analyze epigenetic data both in public and private domains.

## 5 Future work

Single-cell technologies generate large datasets measuring tens of thousands of features over thousands of single cells. Although very efficient to query by genomic region, if we are only interested in a few cells from such large matrices, the EFS library using the Tabix format still has to parse the entire row and filter the columns. Tabix is a commonly used indexing technique for any tabular genomic dataset (the first three columns must be chromosome location, start and end). Interactive analysis, including visualization of these datasets is a challenging task especially for queries to filter by columns (or cells) and efficiently transferring these long matrices between server and client. Piccolo *et al.* (2019) recommend using coordinate based fixed width formats as a fast and scalable approach to query tabular genomic data. In addition, the genomics community has been using HDF5-based formats AnnData or H5ad (Wolf *et al.*, 2018) and loom (https://loompy.org) to store large genomics datasets. We are currently exploring ways to efficiently query HDF5, H5ad or loom format files. These formats do not natively support remote querying like BigWig or Tabix but require an additional server like h5serv (https://support.hdfgroup.org/pubs/papers/RESTful_HDF5.pdf) setup for Web queries.

Recent approaches like Pyranges (Stovner and Sætrom, 2020) have implemented data structures for efficiently manipulating genomic intervals in Python. The EFS library can be extended to incorporate these file formats and data structures to efficiently query, perform interval overlap operations and interactively explore multi-omic datasets.

## 6 Conclusion

Based on the concepts of a NoDB paradigm, we present a file-based Python library, an *in situ* data query system for indexed genomic files, not only for visualization but also transformation. The library implements several features provided by a traditional database system for query, transformation and caching. The use cases demonstrate the flexibility in integrating the EFS library with existing bioinformatic tools and public repositories. We discussed new research approaches to build a comprehensive file-based data visualization and exploration system for genomics datasets.

## Funding

This work was supported by the U.S. National Institutes of Health grant [R01GM114267 and R24MH114815].

*Conflict of Interest*: none declared.
